# Portal, superior mesenteric and splenic vein thrombosis secondary to hyperhomocysteinemia with pernicious anemia: a case report

**DOI:** 10.1186/1752-1947-8-286

**Published:** 2014-08-25

**Authors:** Prashanth Venkatesh, Nissar Shaikh, Mohammad F Malmstrom, Vajjala R Kumar, Bakr Nour

**Affiliations:** 1 Weill Cornell Medical College in Qatar, Ar-Rayyan, Qatar; 2 Department of Anesthesia/ICU and Perioperative Medicine, Hamad Medical Corporation, Doha, Qatar; 3 Department of Radiology, Hamad Medical Corporation, Doha, Qatar; 4 Department of Surgery, Hamad Medical Corporation, Doha, Qatar

**Keywords:** Hyperhomocysteinemia, Laparotomy, Pernicious anemia, Portal vein thrombosis, Thrombophilia markers

## Abstract

**Introduction:**

Acute portomesenteric vein thrombosis is an uncommon but serious condition with potential sequelae, such as small-bowel gangrene and end-stage hepatic failure. It is known to be caused by various pro-thrombotic states, including hyperhomocysteinemia. We describe what is, to the best of our knowledge, the first reported case of concomitant thrombosis of portal, superior mesenteric and splenic veins due to hyperhomocysteinemia secondary to pernicious anemia and no other risk factors.

**Case presentation:**

A 60-year-old Indian man presented with epigastric pain, diarrhea and vomiting. An abdominal imaging scan showed that he had concomitant pernicious anemia and concomitant portal, superior mesenteric and splenic vein thrombosis. A work-up for the patient’s hypercoagulable state revealed hyperhomocysteinemia, an undetectable vitamin B_12_ level and pernicious anemia with no other thrombophilic state. He developed infarction with perforation of the small bowel and subsequent septic shock with multi-organ dysfunction syndrome, and he ultimately died due to progressive hepatic failure.

**Conclusion:**

This report demonstrates that pernicious anemia, on its own, can lead to hyperhomocysteinemia significant enough to lead to lethal multiple splanchnic vein thrombosis. Our case also underscores the need to (1) consider portomesenteric thrombosis in the differential diagnosis of epigastric abdominal pain, (2) perform a complete thrombotic work-up to elucidate metabolic abnormalities that could be contributing to a pro-thrombotic state and (3) initiate aggressive measures, including early consideration of multi-visceral transplantation, in order to avoid decompensation and a significant adverse outcome.

## Introduction

Acute thrombosis of the splanchnic venous circulation is a condition that, albeit rare in the general population, is of vital importance to the clinician, given its life-threatening sequelae, which include mesenteric infarction and progressive liver failure [1]. A variety of etiologies have been shown to cause portal vein thrombosis, such as cirrhosis, infection of the gastrointestinal tract, malignancies and a variety of hypercoagulable states, including genetic deficiencies in coagulation factors as well as acquired thrombophilias, such as anti-phospholipid syndrome and myeloproliferative disease [2]. A high index of suspicion and thorough evaluation are warranted to diagnose portomesenteric infarction in patients with abdominal pain. In addition, a thorough work-up needs to be done in order to elucidate the etiology, especially underlying systemic illness, in those patients diagnosed with portal vein thrombosis. We present what is, to the best of our knowledge, the first reported case of concomitant thrombosis of the portal vein, superior mesenteric vein and splenic vein in a patient with hyperhomocysteinemia secondary to vitamin B_12_ deficiency caused by pernicious anemia.

## Case presentation

A 60-year-old Indian man presented to our institution with complaints of generalized epigastric abdominal pain of moderate intensity for the previous 10 days, with associated nausea, vomiting and diarrhea of recent onset. His past medical history was significant for anemia that required blood transfusions on two separate occasions, but it was otherwise unremarkable. He had arrived in our area from India two weeks prior to presentation. A presumptive diagnosis of acute gastroenteritis was made, and the patient was started on intravenous fluids and admitted to the medical ward.

**Figure 1:**
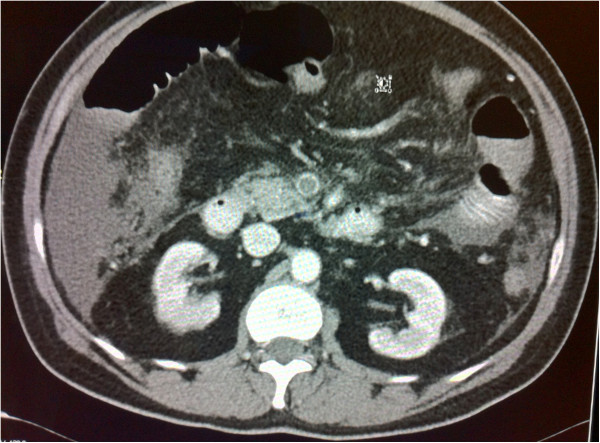
Axial computed tomography scan of the patient’s abdomen with contrast enhancement showing portal vein thrombosis causing complete occlusion of the lumen of the portal vein.

**Figure 2:**
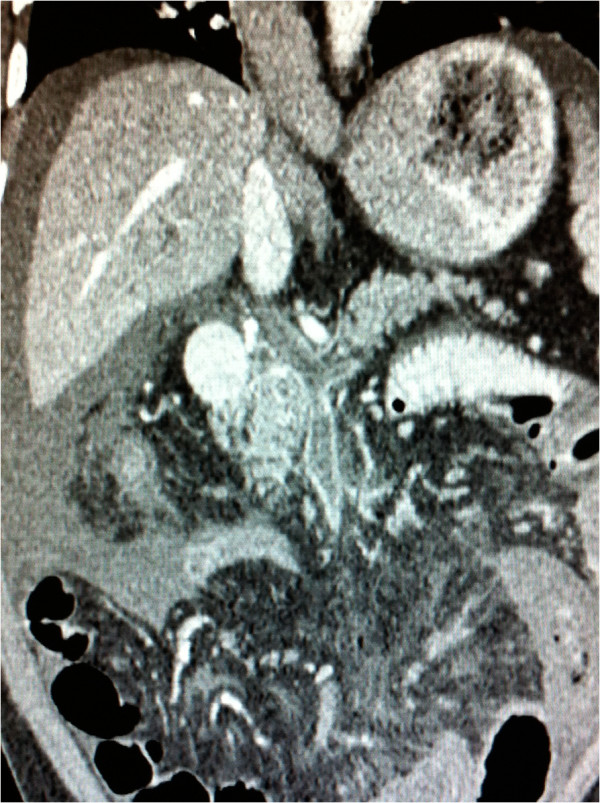
Longitudinal computed tomography scan of the patient’s abdomen with contrast enhancement taken in the axis of the portal vein showing extensive thrombosis involving the superior mesenteric, splenic and portal veins.

Ultrasonography, computed tomography (CT) and magnetic resonance imaging scans of the patient’s abdomen revealed thrombosis of the portal vein, splenic vein and superior mesenteric vein, along with a hypodense region in the liver suggestive of infarction and edematous bowel loops with fat-stranding indicative of mesenteric ischemia (Figures 1 and 2). A preliminary blood work-up revealed macrocytic anemia with 8.7g/dL hemoglobin and 137.1fL mean corpuscular volume. A peripheral blood smear showed numerous macrocytes, macro-ovalocytes and hypersegmented neutrophils indicative of megaloblastic anemia. His serum folate and red blood cell folate levels were normal, but his serum vitamin B_12_ levels were severely low at <44pmol/L. An additional work-up showed a positive 1:80 titer for anti–parietal cell antibody and a markedly positive anti–intrinsic factor antibody level of 131U, thus establishing the diagnosis of pernicious anemia. An extensive coagulation work-up showed a raised prothrombin time (13.3 seconds), an elevated activated partial thromboplastin time (43.4 seconds) and a high international normalized ratio (1.3). It also revealed a normal platelet count, normal protein C and protein S levels, and normal anti–thrombin III function, as well as a negative result for lupus anti-coagulant.

Genetic testing for factor V Leiden mutation and *JAK2* gene mutation was negative. Immunoglobulin G (IgG) and IgM assays for anti-cardiolipin and anti-β_2_-glycoprotein antibodies were below diagnostic values, as were anti-nuclear antibody tests. However, the patient’s serum homocysteine levels were markedly high at 70μmol/L (normal 4μmol/L to 12μmol/L). The results of a test for the methyltetrahydrofolate reductase (*MTHFR*) gene was negative for any remarkable polymorphisms, including the C677T mutation. A genetic test result for factor II mutation was also negative.

**Figure 3:**
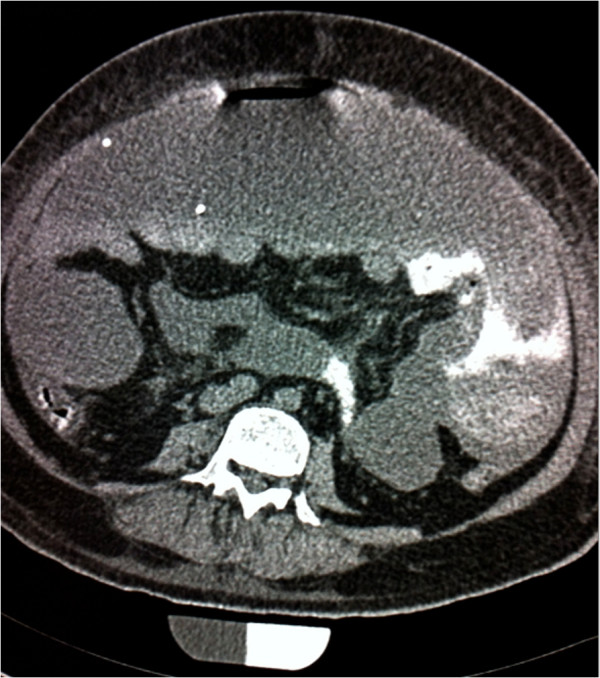
Emergent axial computed tomography scan of the abdomen with contrast enhancement showing extravasation of contrast from the proximal small bowel, indicating bowel perforation.

The patient was admitted to the medical ward and started on anti-coagulation therapy with warfarin and low-molecular-weight heparin, as well as parenteral vitamin B_12_ therapy, after his vein thromboses were deemed surgically incorrigible. During his hospital stay, he developed progressive ascites that required periodic drainage. On day 20 of admission, the ascitic fluid drainage turned turbid and a CT scan showed mesenteric perforation (Figure 3). An emergent laparotomy revealed mesenteric gangrene with perforation, and a bowel resection with ileostomy and mucus fistula creation was performed. The patient’s recovery was complicated by *Bacteroides ovatus* bacteremia and *Klebsiella pneumoniae* sepsis, which resulted in septic shock and multi-organ dysfunction syndrome necessitating intensive care. He eventually developed progressively rising bilirubin and falling albumin levels as well as profound jaundice and encephalopathy, indicative of end-stage hepatic failure. Despite aggressive anti-coagulation therapy being administered continuously throughout the patient’s admission, and in spite of his vitamin B_12_ and homocysteine levels turning normal, his thromboses did not resolve. This resulted in splenic infarction as well as progressive liver failure with severe hepatic encephalopathy. The patient eventually died due to hepatic failure three months into his hospital stay.

## Discussion

Hyperhomocysteinemia is a consequence of vitamin B_12_ deficiency, because vitamin B_12_ is required as a cofactor for the enzyme MTHFR, which remethylates homocysteine to form methionine [3]. Hyperhomocysteinemia has been well-documented to increase the risk of arterial thrombotic events, including myocardial infarction [4], stroke [5] and peripheral arterial disease [6]. Data concerning venous thrombosis also point to an increased risk for hyperhomocysteinemia [3,7-9]. In one case–control study, researchers found an increased risk for venous thromboembolism associated with reduced vitamin B_12_, together with increased methylmalonic acid levels, in the presence of hyperhomocysteinemia [10]. However, the investigators in the cited studies recruited mostly patients with deep vein thrombosis and did not include patients with thrombosis of the splanchnic vasculature.

Patients with portomesenteric vein thrombosis and hyperhomocysteinemia have scarcely been reported. Landman *et al.* reported hyperhomocysteinemia in 12 (28%) of 43 patients with portomesenteric vein thrombosis and concomitant inflammatory bowel disease who were screened for pro-thrombotic conditions [11]. A case series reported by Mushtak *et al*., a group of clinicians at our institution, included a patient with concomitant portal, mesenteric and splenic vein thrombosis associated with hyperhomocysteinemia and vitamin B_12_ deficiency. However, that patient tested negative for anti–intrinsic factor and anti–parietal cell antibodies, and his vitamin B_12_ deficiency was attributed to his long-term vegan diet [12]. The other two patients in their case series also presented with venous thrombosis, hyperhomocysteinemia and vitamin B_12_ deficiency.

The C677T mutation of the *MTHFR* gene has been reported in a patient with pernicious anemia and portomesenteric thrombosis [13]. Our patient, however, tested negative for the same mutation. Kanbay *et al.* reported a case of a patient with elevated liver enzymes, portal vein thrombosis, vitamin B_12_ deficiency and elevated homocysteine levels; however, they did not report a cause of the vitamin B_12_ deficiency [14]. Tan *et al.* reported a case of portal vein thrombosis secondary to hyperhomocysteinemia that led to bowel gangrene, all of which were corrected with folate and vitamin B_6_ supplementation [15]. Other case reports associating portomesenteric vein thrombosis with hyperhomocysteinemia include comorbid folate deficiency [16], factor V Leiden mutation [17], latent essential thrombocythemia [18] and the factor II G20210A mutation [19], with the latter being a relatively common thrombophilia in Caucasian patients but very rare in Asian populations, to which our patient belonged. Our report is unique in that our patient had none of these additional risk factors.

In all of the aforementioned reports of portomesenteric vein thrombosis with vitamin B_12_ deficiency, anti-coagulation with concomitant correction of the nutritional deficiency was undertaken as early as possible, but with varied success. Thus, no clear recommendation was made in those reports regarding the ideal therapy and treatment endpoints for such patients with portal thrombosis secondary to underlying systemic disease.

Our case presented significant management challenges because the three major veins comprising the portal circulation were all acutely thrombosed and deemed surgically incorrigible. Anti-coagulation therapy did not prevent progression to bowel gangrene, which increases mortality to 20% to 50% [20], and ultimately to liver failure. Vitamin B_12_ supplementation was effective in restoring normal vitamin B_12_ and homocysteine levels, but this did not translate to clinical improvement. Anti-coagulation therapy with low-molecular-weight heparin and warfarin is the recommended initial treatment for acute portal vein thrombosis, with a six-month recanalization rate of up to 50% and a complete failure rate of 10% [2]. If this treatment fails, however, injection of thrombolytic agents into the portal vasculature is an option. Recanalization rates are at best modest, and the high rate of complications calls into question the prudence of implementing this therapy [21]. Surgical thrombectomy has been reported, but it carries a high rate of adverse outcomes and is hence not currently recommended. Vascular reconstruction by surgical shunting is an option [1], but it requires patency of the splenic and superior mesenteric veins and hence was not suitable for our patient. Furthermore, this option has been successful only in patients with chronic portal thrombosis. A liver transplant would not have been a viable option for our patient, as the extensive thrombosis of the splanchnic veins would have caused rapid graft failure [22].

The only definitive cure for our patient’s condition would have been multi-visceral transplantation. This would have involved contiguous transplantation of the liver and the small bowel, described previously by Nour *et al*. [22]. The hepatoenteric graft would have been harvested *en bloc* along with the patent splanchnic veins of the donor, and hence would have ensured viability. The patient would have been placed on total parenteral nutrition in the post-operative period until enteral nutrition was adequate [22]. Unfortunately, multi-visceral transplantation was not only unavailable but also infeasible in the face of acute decompensation of the patient’s clinical condition and superimposed sepsis.

## Conclusion

To the best of our knowledge, this is the first reported case of concomitant thrombosis of the portal, superior mesenteric and splenic veins with hyperhomocysteinemia secondary to pernicious anemia and no other risk factors. Prompt and sustained anti-coagulation with vitamin B_12_, together with homocysteine normalization, did not prevent progression to bowel gangrene and liver failure. The dearth of evidence regarding alternatives to anti-coagulation, along with the extensive multi-vessel thrombosis in our patient, limited the treatment options that could be offered. This report highlights the fact that pernicious anemia by itself can lead to hyperhomocysteinemia significant enough to lead to lethal multiple abdominal vein thrombosis. It underscores the need to (1) consider portomesenteric thrombosis in the differential diagnosis of epigastric abdominal pain, (2) perform a complete thrombotic work-up to elucidate metabolic abnormalities that could be contributing to a pro-thrombotic state and (3) initiate aggressive measures, including the early consideration of multi-visceral transplantation, in order to avoid decompensation and a significant adverse outcome.

## Consent

Written informed consent was obtained from the patient’s next of kin for publication of this case report and any accompanying images. A copy of the written consent is available for review by the Editor-in-Chief of this journal.

## Abbreviations

CT: Computed tomography; Ig: Immunoglobulin; MTHFR: Methylenetetrahydrofolate reductase.

## Competing interests

The authors declare that they have no competing interests.

## Authors’ contributions

PV carried out the case review and the literature review and drafted the manuscript. NS carried out the case review and drafted the manuscript. MFM edited and revised the manuscript. VRK acquired and interpreted the radiographic scans. BN edited the manuscript and contributed to the literature review. All authors read and approved the final manuscript.

## References

[B1] Ponziani FR Zocco MA Campanale C Rinninella E Tortora A Di Maurizio L Bombardieri G De Cristofaro R De Gaetano AM Landolfi R Gasbarrini A Portal vein thrombosis: insight into physiopathology, diagnosis, and treatment World J Gastroenterol 2010 16 143 155 10.3748/wjg.v16.i2.143 20066733 PMC2806552

[B2] Parikh S Shah R Kapoor P Portal vein thrombosis Am J Med 2010 123 111 119 10.1016/j.amjmed.2009.05.023 20103016

[B3] Cattaneo M Hyperhomocysteinemia and venous thromboembolism Semin Thromb Hemost 2006 32 716 723 10.1055/s-2006-951456 17024599

[B4] Stampfer MJ Malinow MR Willett WC Newcomer LM Upson B Ullmann D Tishler PV Hennekens CH A prospective study of plasma homocyst(e)ine and risk of myocardial infarction in US physicians JAMA 1992 268 877 881 10.1001/jama.1992.03490070059042 1640615

[B5] Towfighi A Markovic D Ovbiagele B Pronounced association of elevated serum homocysteine with stroke in subgroups of individuals: a nationwide study J Neurol Sci 2010 298 153 157 10.1016/j.jns.2010.07.013 20810133

[B6] Khandanpour N Loke YK Meyer FJ Jennings B Armon MP Homocysteine and peripheral arterial disease: systematic review and meta-analysis Eur J Vasc Endovasc Surg 2009 38 316 322 10.1016/j.ejvs.2009.05.007 19560951

[B7] Ray JG Meta-analysis of hyperhomocysteinemia as a risk factor for venous thromboembolic disease Arch Intern Med 1998 158 2101 2106 10.1001/archinte.158.19.2101 9801176

[B8] Herrmann M Whiting MJ Veillard A Ehnholm C Sullivan DR Keech AC Plasma homocysteine and the risk of venous thromboembolism: insights from the FIELD study Clin Chem Lab Med 2012 50 2213 2219 23093273 10.1515/cclm-2012-0078

[B9] Köktürk N Kanbay A Aydoğdu M Özyılmaz E Bukan N Ekim N Hyperhomocysteinemia prevalence among patients with venous thromboembolism Clin Appl Thromb Hemost 2011 17 487 493 10.1177/1076029610378499 20699251

[B10] Remacha AF Souto JC Piñana JL Sardà MP Queraltó JM Martí-Fabregas J García-Moll X Férnandez C Rodriguez A Cuesta J Vitamin B12 deficiency, hyperhomocysteinemia and thrombosis: a case and control study Int J Hematol 2011 93 458 464 10.1007/s12185-011-0825-8 21475950

[B11] Landman C Nahon S Cosnes J Bouhnik Y Brixi-Benmansour H Bouguen G Colombel JF Savoye G Coffin B Abitbol V Filippi J Laharie D Moreau J Veyrac M Allez M Marteau P on behalf of Groupe d’Etude Thérapeutique des Affections Inflammatoires du Tube Digestif Portomesenteric vein thrombosis in patients with inflammatory bowel disease Inflamm Bowel Dis 2013 19 582 589 10.1097/MIB.0b013e31827eea5f 23385240

[B12] Mushtak A Khan FY AlDehwe B Al-Ani AA Three different presentation of same pathophysiology Acta Inform Med 2012 20 190 191 10.5455/aim.2012.20.190-191 23322977 PMC3508855

[B13] Fernández-Ruiz M Alonso-Navas F Muro E Pérez-Carreras M [Portal and mesenteric vein thrombosis associated with hyperhomocysteinemia and pernicious anemia in a patient heterozygous for the *MTHFR* C677T mutation] [Article in Spanish] Med Clin (Barc) 2011 136 225 226 10.1016/j.medcli.2010.01.011 20207379

[B14] Kanbay M Karakus S Yilmaz U Portal vein thrombosis due to hyperhomocysteinemia caused by vitamin B-12 deficiency Dig Dis Sci 2005 50 2362 2363 10.1007/s10620-005-3064-0 16416191

[B15] Tan K Chow PKH Tan YM Thng CH Portal vein thrombosis secondary to hyperhomocysteinemia: a case report Dig Dis Sci 2006 51 1218 1220 10.1007/s10620-006-8036-5 16944013

[B16] Audemar F Denis B Blaison G Mazurier I Peter A Serbout R [Left branch portal vein thrombosis associated with hyperhomocysteinemia] [Article in French] Gastroenterol Clin Biol 1999 23 1388 1391 10642624

[B17] Famularo G Minisola G Nicotra GC Simone CD Mesenteric and portal vein thrombosis associated with hyperhomocysteinemia and heterozygosity for factor V Leiden mutation World J Gastroenterol 2005 11 7700 7701 16437705 10.3748/wjg.v11.i48.7700 PMC4727217

[B18] Berk DR Ahmed A Portal, splenic and superior mesenteric vein thrombosis in a patient with latent essential thrombocythemia and hyperhomocysteinemia J Clin Gasteroenterol 2006 40 227 228 10.1097/00004836-200603000-00012 16633126

[B19] Marie I Levesque H Le Cam-Duchez V Borg JY Ducrotté P Philippe C Mesenteric venous thrombosis revealing both factor II G20212A mutation and hyperhomocysteinemia related to pernicious anemia Gastroenterology 2000 118 237 238 10.1016/S0016-5085(00)70442-2 10644179

[B20] Kumar S Sarr MG Kamath PS Mesenteric venous thrombosis N Engl J Med 2001 345 1683 1688 10.1056/NEJMra010076 11759648

[B21] Hollingshead M Burke CT Mauro MA Weeks SM Dixon RG Jaques PF Transcatheter thrombolytic therapy for acute mesenteric and portal vein thrombosis J Vasc Interv Radiol 2005 16 651 661 10.1097/01.RVI.0000156265.79960.86 15872320

[B22] Nour B Tzakis AG Abu-Elmagd K Furukawa H Todo S Reyes J Starzl TE Levine BA, Copeland EM3rd, Howard RJ, Sugerman H, Warshaw AL Small bowel transplantation Current Practice of Surgery XIV(6) 1993 New York: Churchill Livingstone 1 15

